# Supplementary figures and images for: Deterministic genetic barcoding for multiplexed behavioral and single-cell transcriptomic studies
Source: eLife. 2025 Feb 5;12:RP88334. doi: 10.7554/eLife.88334 (PMC11798575; doi:10.7554/eLife.88334)

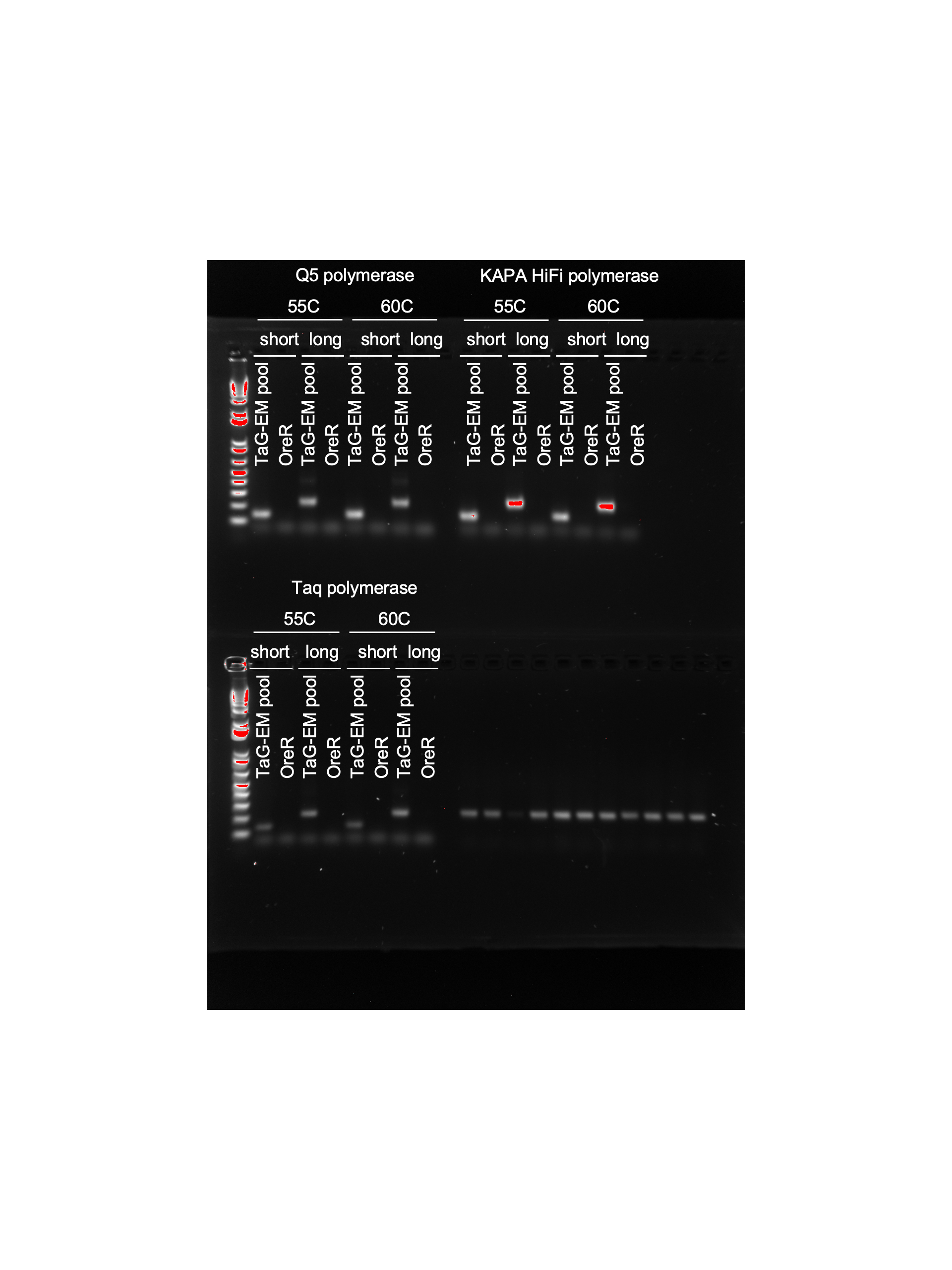

Supplement: Figure 2—figure supplement 1—source data 1. [file elife-88334-fig2-figsupp1-data1.zip › Figure 2 - figure supplement 1 - labelled/UMGC_IL_038_DG_gel_210325_Labeled.tiff]

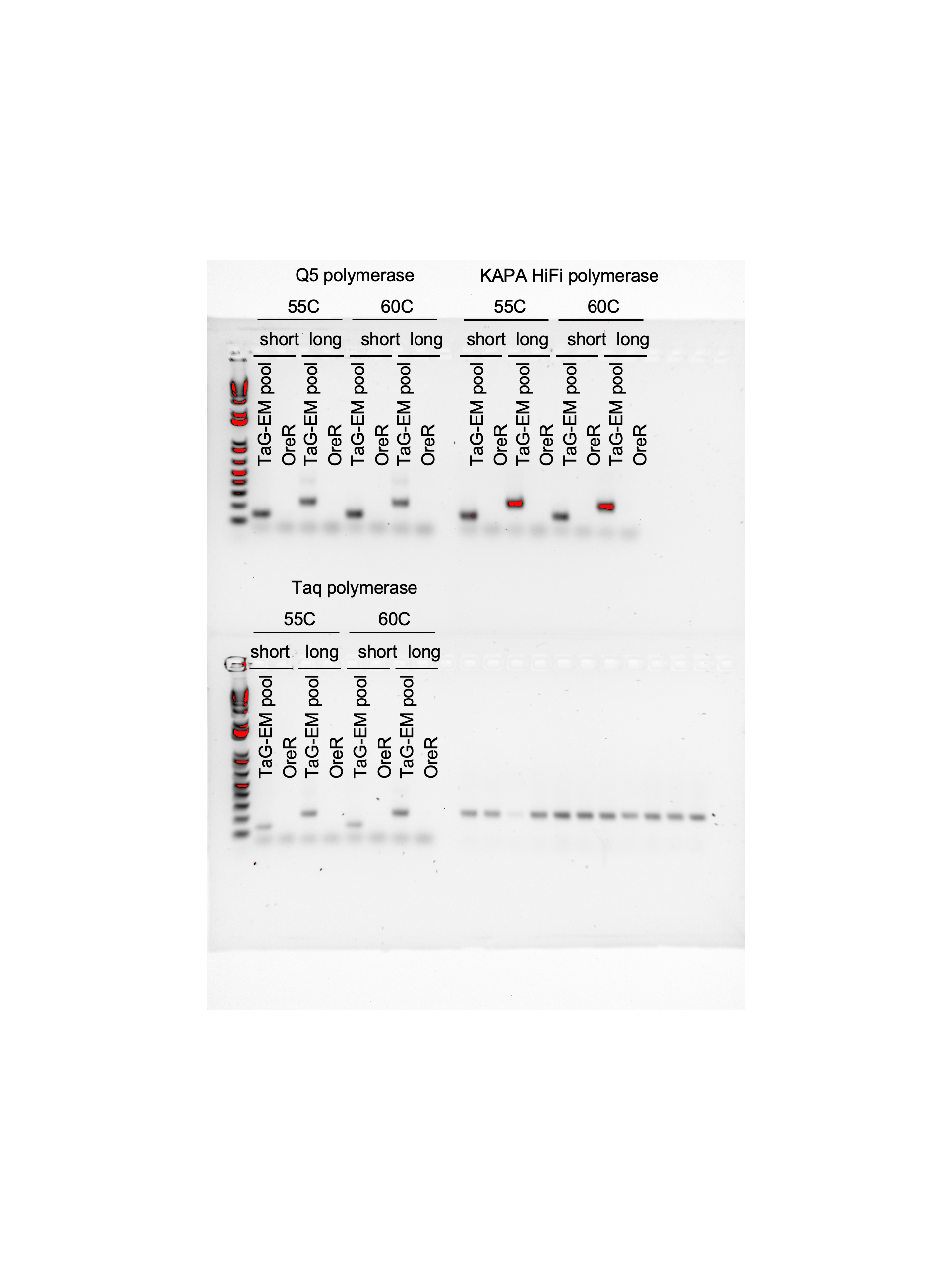

Supplement: Figure 2—figure supplement 1—source data 1. [file elife-88334-fig2-figsupp1-data1.zip › Figure 2 - figure supplement 1 - labelled/UMGC_IL_038_DG_gel_210325i_Labeled.tiff]

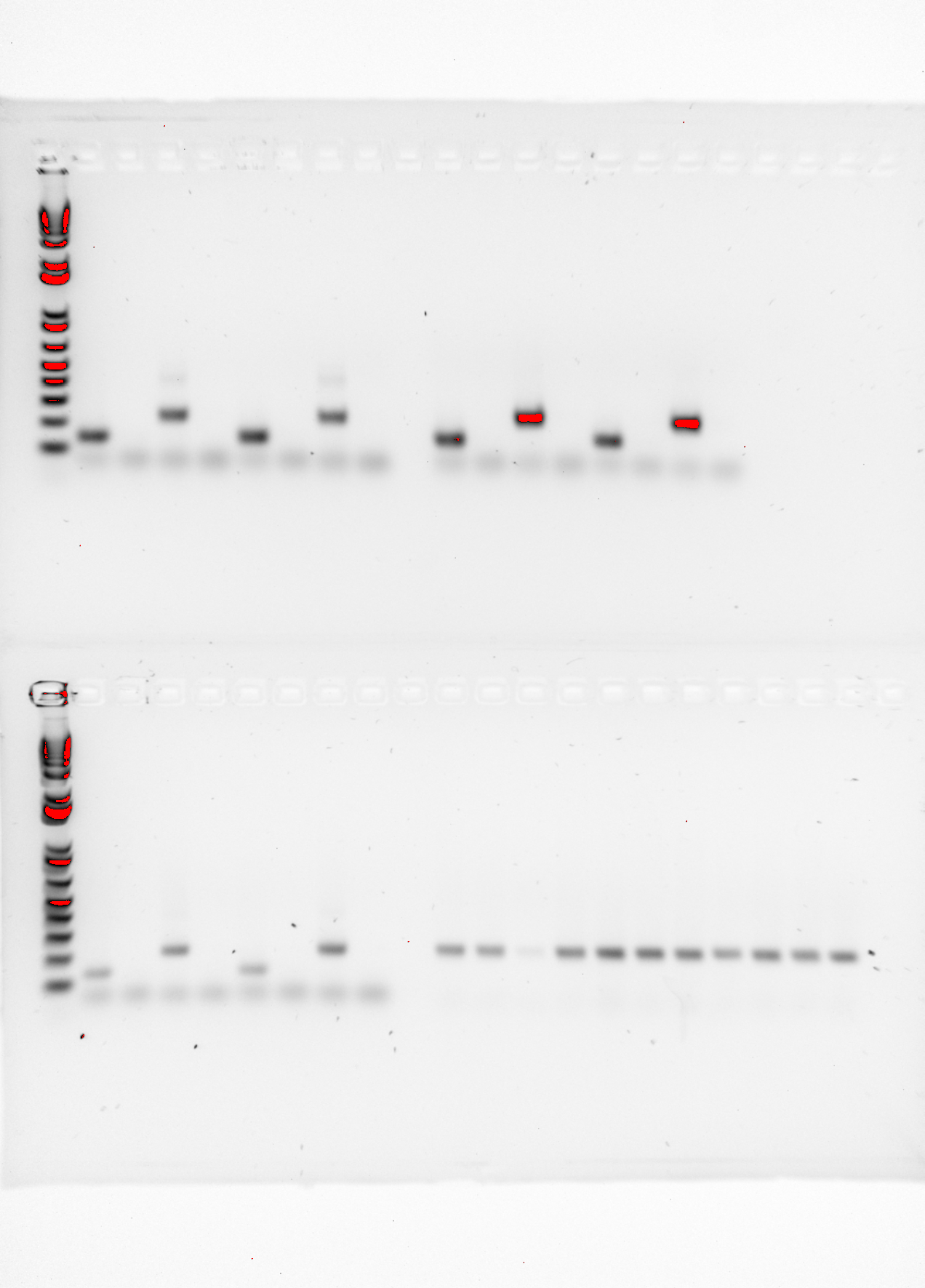

Supplement: Figure 2—figure supplement 1—source data 2. [file elife-88334-fig2-figsupp1-data2.zip › Figure 2 - figure supplement 1 - raw/UMGC_IL_038_DG_gel_210325i.tif]

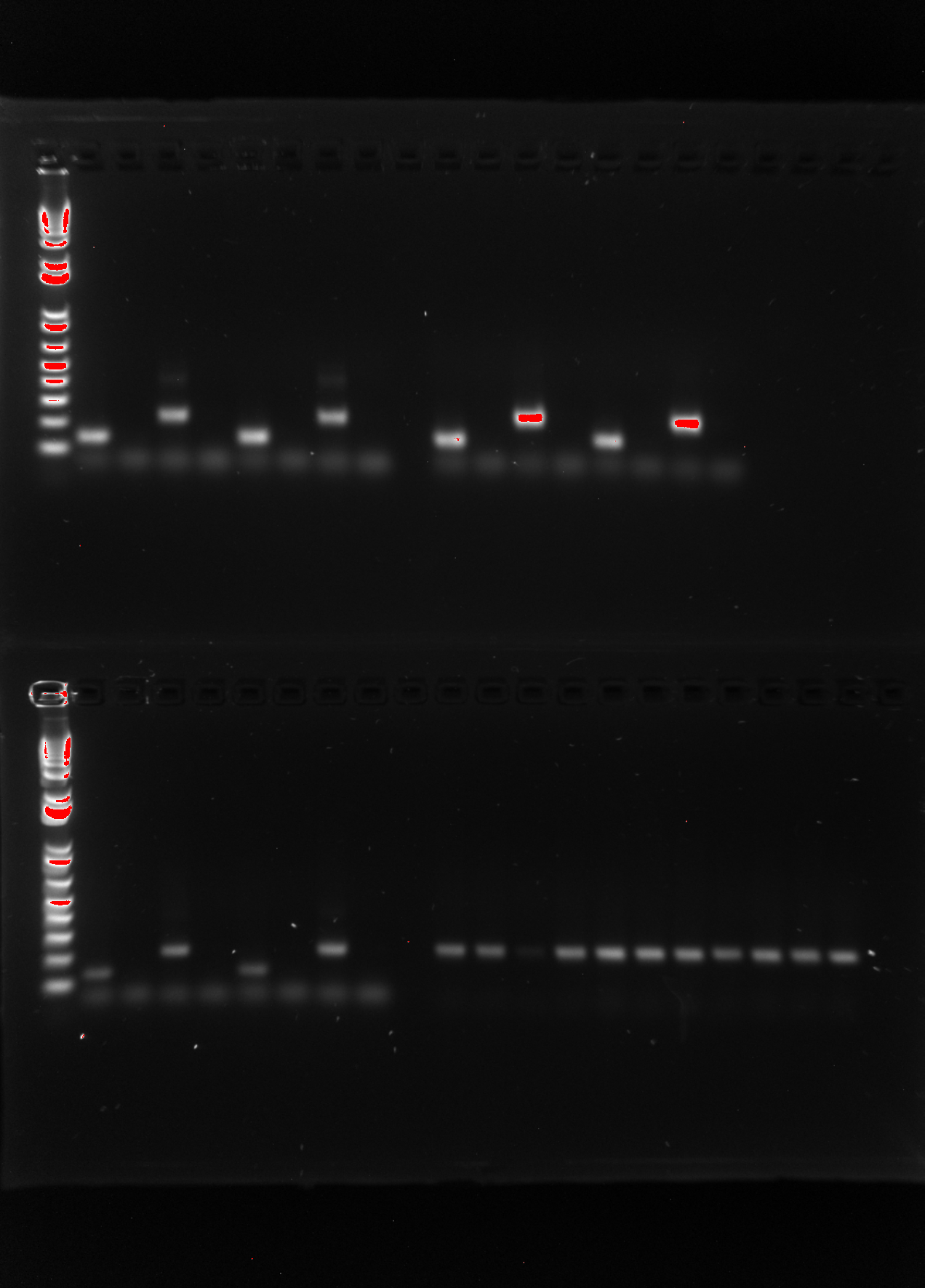

Supplement: Figure 2—figure supplement 1—source data 2. [file elife-88334-fig2-figsupp1-data2.zip › Figure 2 - figure supplement 1 - raw/UMGC_IL_038_DG_gel_210325.tif]
